# The intergenerational transmission of reflective functioning in adoptive families: A prospective study from pre‐adoption to early adolescence

**DOI:** 10.1002/imhj.70089

**Published:** 2026-05-04

**Authors:** Simon Fiore, Patrick Luyten, Bart Soenens, Saskia Malcorps, Karin Ensink, Nicole Vliegen

**Affiliations:** ^1^ Faculty of Psychology and Educational Sciences KU Leuven Leuven Belgium; ^2^ Department of Developmental, Personality and Social Psychology Ghent University Ghent Belgium; ^3^ Research Department of Clinical, Educational and Health Psychology University College London London UK; ^4^ École de psychologie Université Laval Québec Canada

**Keywords:** adoption, intergenerational, parental reflective functioning, protective

## Abstract

Parental reflective functioning is crucial for the development of children's reflective functioning, with cross‐sectional evidence supporting this idea. However, there is a lack of long‐term, prospective studies in this area and no previous studies have examined parental reflective functioning and child reflective functioning in the context of adoption. Using a structural equation modeling approach, this study addressed this knowledge gap by examining the associations between 96 Belgian adoptive mothers’ and fathers’ reflective functioning during the transition to adoptive parenthood, their levels of parental reflective functioning during their adopted child's early childhood, and the children's reflective functioning in early adolescence (*M*_age_ = 12 years, *SD* = .58, range = 11–13; 17 boys, 11 girls). Findings showed that parents’ pre‐adoptive reflective functioning was positively associated with parental reflective functioning in early childhood. Parental reflective functioning in early childhood, but not pre‐adoptive reflective functioning, predicted child reflective functioning in early adolescence. Maternal reflective functioning mediated the association between pre‐adoptive reflective functioning and child reflective functioning. Although the small sample size precludes drawing strong conclusions from this study, this study provides new evidence for the intergenerational transmission of reflective functioning even in the context of adoption. Implications for future research are discussed.

## 1 INTRODUCTION

Parental reflective functioning is the relationship‐specific capacity of parents to understand their child's behavior in terms of underlying mental states, such as thoughts, feelings, intentions, and desires (Slade, 2005). Growing evidence supports the idea that parental reflective functioning acts as a key factor in the development of children's reflective functioning (Midgley et al., 2017), a process referred to as the “intergenerational transmission of reflective functioning” (Rosso & Airaldi, 2016). These processes begin in infancy, when children have not yet developed the ability to regulate their affective states (Campbell et al., 2024), and parental reflective functioning enables parents to accurately and congruently understand and mirror their infant's affective experiences and respond to them in appropriate ways (Fonagy et al., 2018). Parental reflective functioning is associated with a wide variety of socioemotional child outcomes, such as children's attachment security (Fonagy et al., 1991; Zeegers et al., 2017), affect regulation capacities (Heron‐Delaney et al., 2016; Misailidi et al., 2025), and socioemotional adjustment (Moreira et al., 2024). With regard to the development of children's own reflective capacities, numerous studies have shown that parental reflective functioning is positively associated with children's theory of mind, a developmental precursor to reflective functioning that involves the foundational ability to attribute mental states to others to predict or explain behavior (Premack & Woodruff, 1978), from preschool age onward (Malcorps et al., 2023; Nijssens et al., 2021).

The development of children's reflective functioning is underpinned by biological and maturational processes, and is mediated by social experiences within early attachment relationships (Fonagy et al., 2018; Midgley et al., 2017). Processes such as gaze perception and joint attention emerge before the age of 1 year (Del Bianco et al., 2019). Between 3 and 4 years of age, children begin to understand belief–desire reasoning, allowing them to predict others’ behavior (Wertz & German, 2007). Between the ages of 4 and 5 years, children develop the ability to understand false beliefs, that is, the capacity to understand that another person's beliefs can differ from reality (Wellman et al., 2001). Beyond early childhood, children's capacities to understand the mental states of others, and themselves, continue to develop in complexity, supported by further development in attentional capacities and executive functioning (Bosacki, 2016). From middle childhood, children increasingly understand the constructivist nature of mental processes, and attribute active and dynamic mental states to themselves and others to explain observable behavior (Weimer et al., 2017).

**Key Findings**

- Pre‐adoptive reflective functioning predicts parental reflective functioning in adopted children's early childhood.
- Parental reflective functioning in adopted children's early childhood predicts child reflective functioning in early adolescence.
- Older child age at placement attenuates the association between maternal reflective functioning in early childhood and child reflective functioning in early adolescence.

**Statement of Relevance**

This study directly investigated the intergenerational transmission of reflective functioning by prospectively examining the relationship between parental reflective functioning prior to transnational adoption, parental reflective functioning in their adopted children's early childhood, and adopted children's reflective functioning in early adolescence. This study contributes to research on the stability of reflective functioning in the transition to parenthood and the intergenerational transmission of reflective functioning, thereby informing both prevention and intervention strategies.

The understanding of mental processes in adolescence becomes more sophisticated, with a greater emphasis on understanding social dynamics and the capacity to predict others’ behavior in ambiguous and complex social situations (Białecka‐Pikul et al., 2017). This profound increase in complexity is supported by neurobiological changes that occur in adolescence and by the growing complexity of social dynamics within peer groups (Blakemore, 2018). Reflective functioning, as described in the current literature, becomes more prominent from middle childhood onward, as children develop a greater ability to integrate their cognitive and emotional understanding of how thoughts, feelings, and intentions interact within the social dynamics of close relationships (Ensink & Mayes, 2010; Luyten et al., 2020). Reflective functioning allows children to navigate social relationships effectively, and studies have increasingly shown that impairments in this capacity are associated with socioemotional difficulties (Ensink & Mayes, 2010; Luyten et al., 2020). Among adolescents, impairments in reflective functioning have been found to confer a risk for internalizing difficulties (Martin‐Gagnon et al., 2023), externalizing difficulties (Martin‐Gagnon et al., 2023), high levels of impulsivity, and maladaptive emotion‐regulation difficulties (Kahya & Munguldar, 2022). Moreover, adolescence has been found to be a developmental period in which personality difficulties characterized by impairments in reflective functioning, including borderline personality disorder, have their onset (Sharp et al., 2018). Therefore, a nuanced understanding of the development of reflective functioning, and its intergenerational transmission, is warranted.

There is increasing cross‐sectional evidence for the association between parents’ reflective capacities and their children's theory of mind, an important component of reflective functioning, in early childhood (Malcorps et al., 2023; Nijssens et al., 2021), and more directly between parental and child reflective functioning as demonstrated by studies in samples of community adolescents (with correlations ranging from *r* = .38 to *r* = .79) (Benbassat & Priel, 2012; Rosso & Airaldi, 2016), school‐aged children with histories of sexual abuse (with correlations ranging from *r *= .31 to *r *= .33) (Ensink et al., 2015), and children with type 1 diabetes (*r *= .79) (Costa‐Cordella et al., 2021). However, there is a lack of longitudinal evidence on these associations. The London Parent–Child Project has provided some seminal evidence on the development of reflective functioning and its longitudinal relationship with parental attachment security, assessed during pregnancy (Fonagy et al., 1991). In particular, studies from this project have shown maternal attachment security to be significantly associated with children's understanding of emotion several years later (Steele & Steele, 2005) and with adolescents’ reflective functioning, as coded on the Adult Attachment Interview (*r* = .37) (Steele et al., 2016). However, it is generally agreed that attachment and reflective functioning are loosely coupled and that parents with secure attachment may differ from one another in terms of reflective functioning (Sharp & Fonagy, 2008). Therefore, the longitudinal association between parents’ reflective capacities and their children's reflective functioning remains to be investigated. Further indirect evidence is obtained in the domain of parental mind‐mindedness, the parents’ capacity to perceive their young children as individuals with minds of their own, which has been shown to have longitudinal associations with the development of children's theory of mind (Fishburn et al., 2022; Meins et al., 2002). However, these associations have not yet been examined longitudinally in the context of parental reflective functioning. Furthermore, the role of parents’ reflective capacities in the development of children's reflective capacities in early adolescence remains unclear, requiring future research.

The context of transnational adoption provides a particular framework for studying the intergenerational transmission of reflective functioning for two reasons. First, as transnationally adopted children often have complex histories of adversity prior to adoption, including understimulation, neglect, and abuse (Jiménez‐Morago et al., 2015), they are at increased risk for socioemotional difficulties (Brodzinsky et al., 2022; Fiore et al., 2025). As reflective functioning has been identified as a transdiagnostic factor involved in a wide range of socioemotional difficulties (Luyten et al., 2020), understanding the role the reflective capacities of adoptive parents may have in the development of this factor could offer valuable insights. Second, although adoptive parenthood shares similarities with non‐adoptive parenthood, it also entails several unique characteristics and complexities (Fiore et al., 2024). For instance, adoptive parents have often undergone rigorous preadoption screening and training to ensure high standards of care and reflective skills before receiving permission to adopt (Stroobants et al., 2011; Tang & Vliegen, 2014). Nevertheless, extant literature describes a period of destabilization that can follow adoption, sometimes referred to as a period of post‐adoption “blues” (Foli & Thompson, 2004; Foli et al., 2017, 2014). In this context, the potentially challenging nature of children's socioemotional functioning—often related to adversity experienced in their early lives—may contribute to this destabilization (Foli et al., 2012; Viana & Welsh, 2010). Therefore, the transition to adoptive parenthood provides a unique opportunity to examine the stability of reflective functioning in a period of profound emotional adjustment to a new family situation.

### 1.1 The present study

This is the first longitudinal investigation of the intergenerational transmission of reflective functioning over a period of more than 10 years, offering a robust test of this process.

The present study had three main aims: (1) to examine whether pre‐adoptive reflective functioning of adoptive mothers and fathers predicted parental reflective functioning in early childhood; (2) to investigate whether parental reflective functioning mediated the relationship between pre‐adoptive reflective functioning and adopted children's reflective functioning in adolescence; and (3) to investigate whether parents’ and children's reflective functioning predicted internalizing and externalizing difficulties in adolescence. We hypothesized that (1) pre‐adoptive reflective functioning would be positively associated with parental reflective functioning in early childhood, (2) parental reflective functioning would predict child reflective functioning and mediate the association between pre‐adoptive reflective functioning and child reflective functioning, and (3) both parental and child reflective functioning would be inversely associated with socioemotional adolescent difficulties.

## 2 METHODS

### 2.1 Participants and procedure

The sample consisted of 48 adoptive families in early childhood (i.e., adoptive mother, father and child), participating in the Leuven Adoption Study, an ongoing longitudinal project on the socioemotional development of transnationally adopted children and their adoptive parents, in which trained research assistants have conducted annual home visits since 2009 including observations, interviews, structured tasks, and completion of questionnaires (Malcorps et al., 2022, 2023, 2021). Families were initially contacted via adoption agencies, social media, and meetings for prospective adoptive parents. At the time of the pre‐adoption interview, adoptive mothers (*M*_age_ = 33.10 years, *SD *= 3.32, range 27–42) and adoptive fathers (*M*_age_ = 34.29 years, *SD* = 3.80, range 27–46) had participated in extensive psychosocial screening procedures provided by the Belgian agency for Public Health, Welfare, and Family to be approved by the Central Authority for Adoption. All parents were Belgian, in heterosexual relationships (*M*_duration_ = 10.83 years, *SD* = 3.56, range 4.25–20.41), spoke Flemish as their first language, and had no biological children of their own. Regarding education, 87.5% of adoptive mothers had attained higher education (47.92% with a bachelor's degree, 39.58% with a master's degree), as had 70.83% of adoptive fathers (35.72% with a bachelor's degree, 35.11% with a master's degree). The average time between the first interview and the second interview (which was conducted 6 months after the child's arrival) was 10.68 months (*SD* = 5.11, range = 3–29).

The sample of children adopted in early childhood consisted of 34 boys (70.8%) and 14 girls (29.2%), with an average age of 13.43 months at placement (*SD* = 6.56, range = 4–30 months). Children were adopted from eight countries: Ethiopia (27), South Africa (10), Kazakhstan (6), Burkina Faso (1), China (1), Nigeria (1), Sri Lanka (1), and Uganda (1). On average, children had lived in 2.35 different placements before being adopted (*SD* = .83, range = 1–5). Of the initial 48 families, 37 participated when the child was in early adolescence, resulting in an attrition rate of 33%. However, five adolescents declined to participate in the interview but expressed interest in future home visits, and four had not yet reached early adolescence at the time of the present study. As a result, the sample in early adolescence consisted of 28 adolescents (*M*_age _= 12 years, *SD* = .58, range = 11–13), comprising 17 boys and 11 girls.

### 2.2 Measures

#### 2.2.1 Adoption Expectations Interview

The Adoption Expectations Interview (AEI) (Luyten et al., 2008) is a semi‐structured interview consisting of 20 questions that takes approximately 1.5 h to administer. The AEI is specifically tailored to experiences of the adoption process and expectations about upcoming adoptive parenthood, which has been validated (Malcorps et al., 2021), and includes questions about (1) the decision to adopt and the feelings related to the adoption procedure; (2) current fantasies and feelings about the child, and what the parent thinks the child will need immediately after child placement; (3) how prospective parents imagine themselves and their partner in their parental role; and (4) how they imagine their child's future life (Luyten et al., 2008). The interview contains several probes to engage parents in reflecting upon their own or their future child's mental states, for example: “*How did that make you feel*?”. Each parent was interviewed individually by a research assistant, and pre‐adoptive reflective functioning was coded by two certified scorers who showed excellent inter‐rater reliability (intraclass correlation coefficient = .70–1).

#### 2.2.2 Working Model of the Child Interview

The Working Model of the Child Interview (WMCI) (Zeanah et al., 1986) is a semi‐structured interview consisting of 16 questions designed to assess a parent's representations of their child, and the child's personality and actual behavior in the here‐and‐now parent–child relationship. Interviews were individually administered to adoptive mothers and fathers 6 months after child placement by trained research assistants and took approximately 45 min to administer. An adapted version of the WMCI devised in collaboration with the developers of the Parent Development Interview (Schechter et al., 2008; Slade & Sleed, 2024) includes additional probes designed to elicit parental reflective functioning, allowing the narratives to be coded using the Reflective Functioning Scale (RFS) (Fonagy et al., 1998). For the purposes of this study, the questions of the WMCI were slightly adapted for adoptive parents. For example, whereas the original WMCI includes questions about pregnancy, adoptive parents were asked about their initial reactions to meeting their child. Parental reflective functioning was coded by two scorers, one of whom was a certified coder. Inter‐rater reliability between the two scorers was excellent (intraclass correlation coefficient = .79).

#### 2.2.3 Child Attachment Interview

The Child Attachment Interview (CAI; Target et al., 1998) is a semi‐structured interview consisting of 17 questions that takes approximately 45 min to administer. It was developed to assess the child's self‐representations and their representations regarding their primary attachment figures, particularly in the context of recent experiences of conflict, hurt, illness, distress, separation, and loss (Target et al., 2003). Two additional adoption‐specific questions were included for this study sample in consultation with Yael Shmueli‐Goetz, one of the developers of the CAI: (1) “*When did you first discuss your adoption with your parents?*” or, in the event that the adolescent could not recall, “*Can you recall the last time you spoke with your parents about your adoption?*”; and (2) “*When was the last time you thought about your birth mom/birth dad?*”. Child reflective functioning was coded by the first author, who was trained by Karin Ensink, the developer of the Child Reflective Functioning Scale (CRFS; Ensink & Oandasan, 2013). A subset of 10 interviews was independently double coded with Karin Ensink, with all discrepancies resolved through detailed discussion until full consensus was achieved.

#### 2.2.4 Reflective Functioning Scale

The RFS (Fonagy et al., 1998; Slade & Sleed, 2024) is a gold standard tool for coding reflective functioning in interview transcripts, following a standardized coding procedure in order to assess markers of parental reflective functioning, including (a) awareness of the nature of mental states, (b) explicit efforts to uncover the mental states underlying behavior, (c) recognition of developmental aspects of mental states, and (d) consideration of mental states in relation to the interviewer (Fonagy et al., 1998). An overall reflective functioning score is derived based on the analysis of these markers, ranging from –1 (negative) to 9 (exceptional), with scores classified as negative to low (–1 to 3), borderline (4), and average to high (5 to 9). The RFS has been widely applied and validated with various interview formats, including the Pregnancy Interview (Slade, 2003), the Parent Development Interview (Slade & Sleed, 2024), the WMCI (Zeanah et al., 1986), and the AEI (Luyten et al., 2008).

#### 2.2.5 Child Reflective Functioning Scale

Child reflective functioning was coded from their responses to the CAI using the CRFS, which is an adaptation from the RFS (Ensink & Oandasan, 2013). The CRFS evaluates children's capacity to articulate their own and others’ internal experiences while describing current or past interactions with their caregivers. The manual provides detailed descriptions and examples of varying levels and types of reflective functioning. Similar to the RFS, narratives are rated on an 11‑point scale (–1 to 9), anchored at six points to capture the extent to which children interpret interpersonal interactions and personal reactions in terms of mental states.

#### 2.2.6 Internalizing and externalizing difficulties

Adoptive parents and adolescents individually completed the Strengths and Difficulties Questionnaire (SDQ; Goodman, 1997) when children were on average 12 years old, at the same assessment wave during which the CAI was administered. The SDQ includes 25 items rated on a 3‐point Likert scale (0 = *not true*, 1 = *somewhat true*, 2 = *certainly true*) divided into five 5‐item scales that focus on prosocial behaviors, emotional symptoms, conduct problems, hyperactivity/inattention, and peer problems. To derive broadband scale scores, the emotional symptoms and peer problems were combined into an internalizing scale (e.g., “*I am often unhappy, down‐hearted or tearful*”) and hyperactivity/inattention and conduct problems were combined into an externalizing scale (e.g., “*I get very angry and often lose my temper*”) (Kulawiak et al., 2020). Satisfactory reliability and construct and convergent validity of both broadband scales in clinical and non‐clinical adolescent samples have been widely documented (Essau et al., 2012; Vugteveen et al., 2021). In the current sample, Cronbach's alphas for internalizing difficulties were .73 for maternal reports, .78 for paternal reports, and .67 for adolescent reports; for externalizing difficulties, they were .86 for maternal reports, .83 for paternal reports, and .76 for adolescent reports.

### 2.3 Statistical analyses

Preliminary analyses were conducted using SPSS Statistics 30. Specifically, means and standard deviations were calculated for all main study variables (pre‐adoptive reflective functioning, parental reflective functioning, child reflective functioning, and internalizing and externalizing difficulties) and demographic variables. Next, paired‐sample *t*‐tests were conducted to investigate possible differences between adoptive mothers, fathers, and adolescents, and independent‐sample *t*‐tests were used to investigate differences between adopted boys and girls for the main study variables. Finally, Pearson correlations were calculated between the main study variables and demographic variables (e.g., parental education).

**FIGURE 1:**
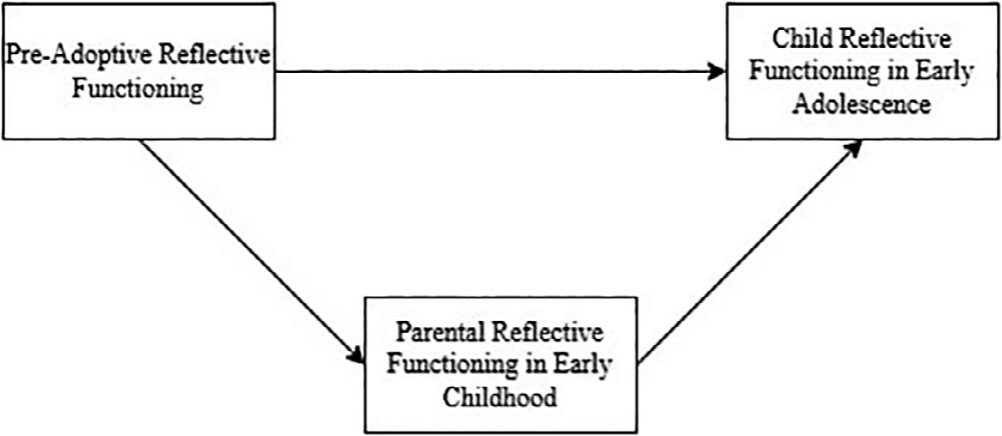
Conceptual representation of the mediation model.

For the primary analyses, a structural equation modeling approach in Mplus version 8.8 was used (Muthén & Muthén, 2017). For Hypothesis 1 and Hypothesis 2 two different structural equation mediation models were conducted, one for maternal reflective functioning and one for paternal reflective functioning, constructed in three different steps. First, a direct effect model was conducted in which pre‐adoptive reflective functioning directly predicted child reflective functioning in early adolescence. Second, parental reflective functioning in the adopted child's early childhood was included as a mediator, thereby testing the indirect path from pre‐adoptive reflective functioning to child reflective functioning. Third, the direct path from pre‐adoptive reflective functioning to child reflective functioning was included to test the direct and indirect paths simultaneously (Figure 1). Finally, to determine whether the full or partial mediation model fitted the data best, the Satorra–Bentler scaled‐difference chi‐square test (ΔSBS‐*χ* ^2^) was used (Satorra & Bentler, 2001). To investigate Hypothesis 3, a multivariate multiple regression analysis was conducted to determine whether child reflective functioning in early adolescence predicted socioemotional difficulties. Additionally, to investigate whether parents’ reflective capacities predicted socioemotional difficulties in adolescence, internalizing and externalizing difficulties were included as separate outcomes in the mediation models.

Model fit for each model was evaluated with the ratio of chi‐square/degrees of freedom, comparative fit index, and root mean square error of approximation, with respective values around 2 or lower, .95 or higher, and around .08 indicating good model fit (Hu & Bentler, 1999). To control for the false discovery rate in the mediation models used for Hypothesis 3, the Benjamini–Hochberg procedure was applied at a significance level of *α* = .05 (Thissen et al., 2002).

Finally, in a set of sensitivity analyses, child age at placement was investigated as a moderator in the maternal and paternal mediation models, as a proxy of adversity prior to adoption (Fiore et al., 2025; Julian, 2013). More specifically, child age at placement was analyzed both continuously (with interaction effects) and categorically (using 12 months as a cut‐off), based on evidence of increased risk of socioemotional difficulties for children adopted after 12 months of age (Van den Dries et al., 2009). Furthermore, given the modest sample size, the statistical power of the mediation models was investigated using a Monte Carlo post hoc power analysis approach.

## 3 RESULTS

### 3.1 Preliminary analyses

Descriptive statistics for the main study variables and demographic variables are provided in Table 1. Paired‐sample *t*‐tests indicated that adoptive mothers scored higher in terms of reflective functioning than adoptive fathers, both prior to adoption and in their adopted child's early childhood, representing small to medium effect size differences. Levene's tests indicated no significant differences between adoptive mothers’ and fathers’ distributions of pre‐adoptive reflective functioning (F(1,95) = .04, *p* = .844) and parental reflective functioning (F(1,93) = .14, *p* = .709).

**TABLE 1:** Dependent *t*‐test comparisons for adoptive mothers and fathers.

Variable	Adoptive mothers				Adoptive fathers				Mean difference			
	*M*	*SD*	Min	Max	*M*	*SD*	Min	Max	*t*‐statistic	*df*	*p*	Cohen's *d*
Pre‐adoptive RF	5.76	1.03	4	8	5.28	1.14	3	8	2.75	46	.009^**^	.40
PRF early childhood	5.32	1.21	3.5	8	4.16	1.33	2	7	4.46	45	˂.001^***^	.67
Internalizing difficulties	3.08	1.66	2	9	3	1.41	2	7	.25	24	.808	.05
Externalizing difficulties	5.76	2.20	3	10	6.32	2.06	3	10	−1.53	24	.139	–.31

Abbreviations: PRF, parental reflective functioning; RF, reflective functioning.

** *p* < .01.

*** *p* < .001.

Interestingly, paired‐sample *t*‐tests indicated that reflective capacities decreased significantly from pre‐adoption to early childhood among both adoptive mothers (*t*(44) = 2.84, *p *= .007, *d *= .42) and adoptive fathers (*t*(46) = 5.66, *p* < .001, *d *= .83). Despite a decrease in reflective functioning scores, very few adoptive mothers scored below the “borderline” threshold of 4. In contrast, the proportion of adoptive fathers scoring below this threshold increased substantially from pre‐adoption to early childhood. When their adopted child was in early childhood, 8.9% of mothers and 36.2% of fathers scored below the borderline score of 4, indicating mentalizing in which links between mental states, or between mental states and behavior, were rudimentary. Additionally, the majority of adoptive mothers demonstrated better than ordinary reflective functioning. At pre‐adoption, 66% of adoptive mothers scored above 5, and this proportion decreased to 46.7% during early childhood. For adoptive fathers, 46.8% scored above 5 pre‐adoption, but only 23.4% did so in early childhood.

To examine the stability of reflective functioning scores from pre‐adoption to post‐adoption, change scores were calculated and categorized into three groups: stable or minimal change (change < 1 point on the RFS), decrease of ≥1 point on the RFS, and increase of ≥1 point. Among adoptive mothers, 42.2% showed stable scores or minimal change, 42.3% showed a decrease of ≥1 point (with 15.6% decreasing by 2 or more points), and 15.5% demonstrated an increase of ≥1 point. In contrast, 34.0% of adoptive fathers exhibited stable or minimal change, 59.6% showed a decrease of ≥1 point (with 36.2% decreasing by 2 or more points), and 6.4% showed an increase of ≥1 point. Although these descriptive trends suggested that adoptive fathers were more likely to experience declines in reflective functioning, while adoptive mothers were more likely to demonstrate increases, these categorical differences did not reach statistical significance, *χ* ^2^(2, *N* = 92) = 3.54, *p* = .170.

Child reflective functioning, as assessed with the CRFS (11‐point scale), averaged 3.10 (*SD* = 2.41) among early adolescent adoptees, corresponding to a relatively low, or “banal”, level of reflective functioning. Girls showed a higher mean score (*M* = 4.20, *SD* = 2.94) than boys (*M* = 2.69, SD = 1.92), but this difference was not statistically significant, likely due to the large variability in girls’ scores (Table 2). This heterogeneity, combined with the modest sample size, may have limited the statistical power to detect sex differences.

**TABLE 2:** Independent *t*‐test comparisons for transnationally adopted boys and girls.

Variable	Boys		Girls		Mean difference		
	*M*	*SD*	*M*	*SD*	*t*‐statistic	*p*	Cohen's *d*
Child reflective functioning	2.69	1.92	4.20	2.94	−1.32	.207	–.56
Internalizing difficulties M	2.40	.51	4.10	2.23	−1.99	.059†	–.81
Internalizing difficulties F	2.87	1.55	3.20	1.23	–.71	.487	–.29
Internalizing difficulties A	4.40	2.92	2.00	2.71	2.07	.05*	.85
Externalizing difficulties M	6.00	2.45	5.40	1.84	.50	.621	.20
Externalizing difficulties F	6.40	1.88	6.20	2.39	.00	1	.00
Externalizing difficulties A	7.67	2.72	7.60	4.97	.04	.970	.02

Abbreviations: A, adolescent report; F, father report; M, mother report.

† < .0

* < .0

Regarding adolescent socioemotional difficulties, most adoptive parents and adolescents reported that socioemotional difficulties fell within the normal range (66.7%–74.1% for internalizing and 51.9%–59.3% for externalizing difficulties), using age‐ and gender‐specific normative data from the SDQ (Vugteveen et al., 2022). However, a substantial group reported marked difficulties, particularly in relation to externalizing difficulties. Specifically, 29.6% of adoptive mothers and 25.9% of adoptive fathers rated their child's externalizing difficulties in the abnormal range, whereas 33.0% of adolescents self‐reported externalizing scores in this range. For internalizing difficulties, 7.4% of adoptive mothers and 15.4% of adoptive fathers scored their child in the abnormal range, compared with 14.8% of adolescents’ self‐reports. Adoptive mothers and fathers did not differ significantly in their perceptions of internalizing or externalizing difficulties (all *p*s > .05). Boys reported significantly more internalizing difficulties compared with girls (Table 2).

Pearson bivariate correlations between study variables are presented in Table 3. For adoptive mothers and fathers, pre‐adoptive reflective functioning was positively correlated with parental reflective functioning when their child was in early childhood. Furthermore, maternal reflective functioning in early childhood was significantly correlated with child reflective functioning (*r* = .45, *p* < .05). No such associations were found for adoptive fathers. Finally, parental education and child sex showed trends toward significance with the study variables and were therefore included as covariates.

**TABLE 3:** Correlations among study variables for adoptive mothers and fathers separately.

Variables	1	2	3	4	5	6	7	8	9
1. Pre‐adoptive reflective functioning		.56^**^	.31^†^	–.03	–.30	–.11	.24	.04	.13
2. Parental reflective functioning	.39^*^		.45^*^	.10	–.09	.00	.11	.03	–.21
3. Child reflective functioning	.13	.16		.30	.05	.25	.27	.01	.11
4. Internalizing difficulties	.33	.35^†^	.14		.40^*^	.04	.38^†^	–.01	–.08
5. Externalizing difficulties	–.07	.08	–.17	.18		–.09	–.10	.27	–.28
6. Child age at placement	.11	.45^*^	.25	–.02	–.16		.21	–.35^†^	–.12
7. Child sex	.18	–.21	.27	.15	.00	.21		–.34^†^	.07
8. Parental education	.37^†^	.37^†^	.23	.14	.28	–.13	–.12		–.00
9. Parental age at child placement	–.07	–.07	.20	.08	–.18	–.00	–.08	–.06	

*Note*: Maternal correlations are depicted above the diagonal, paternal correlations below the diagonal.

^†^ *p* < .10.

* *p* < .05.

** *p* < .01.

### 3.2 Primary analyses

#### 3.2.1 The predictive role of maternal reflective functioning

**FIGURE 2:**
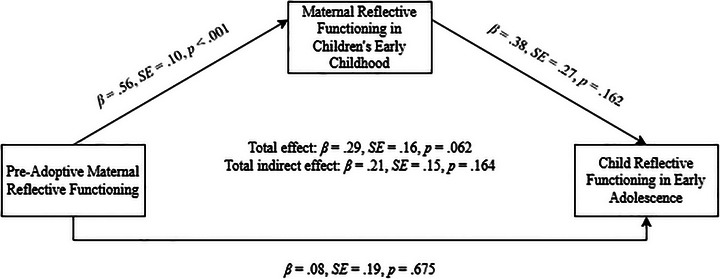
Final maternal mediation model.

The models for maternal reflective functioning showed excellent fit; the full results are presented in Table 4. In Step 1, the direct effect of pre‐adoptive maternal reflective functioning on child reflective functioning showed a trend toward significance (*β* = .28, *SE* = .16, *p* = .080). In Step 2, pre‐adoptive maternal reflective functioning significantly predicted maternal reflective functioning in the adopted child's early childhood (*β* = .56, *SE* = .10, *p* < .001), which in turn significantly predicted child reflective functioning in adolescence (*β* = .44, *SE* = .22, *p* = .046). The indirect effect was also significant (*β* = .24, *SE* = .13, *p* = .050). In Step 3, the direct paths from pre‐adoptive maternal reflective functioning to child reflective functioning and from maternal reflective functioning to child reflective functioning were nonsignificant (*β* = .08, *SE* = .19, *p* = .675; *β* = .38, SE = .27, *p *= .162) (Figure 2). The total indirect effect was nonsignificant (*β* = .21, *SE* = .15, *p* = .164), but the total effect approached significance (*β* = .29, *SE* = .16, *p* = .062). Finally, a model comparison showed no significant improvement with the inclusion of the direct path (ΔSBS‐*χ* ^2^(1) = .19, *p* = .664).

**TABLE 4:** Mediation model of maternal reflective functioning.

	Predictor Pre‐adoptive RF			Mediator Maternal RF			Outcome Child RF		
	*β* (*SE*)	95% CI	*p*‐value	*β* (*SE*)	95% CI	*p*‐value	*β* (*SE*)	95% CI	*p*‐value
**Step 1**									
Pre‐adoptive RF							.28 (.16)		.076
Child gender^a^	.75 (.36)	[.05; 1.45]	.037^*^				.56 (.37)	[–.11; 1.24]	.133
Maternal education	.18 (.25)	[–.31; .66]	.481				–.01 (.03)	[–.33; .45]	.846
Time between assessments	–.03 (.02)	[–.07; .01]	.187				.09 (.22)	[–.07; .06]	.676
*R* ^2^	.12 (.10)		.240				.19 (.14)		.186
**Step 2**									
Pre‐adoptive RF				.56 (.10)		.000^***^			
Maternal RF							.44^*^ (.22)	[.01; .87]	.046^*^
Child gender^a^	.75 (.36)	[.05; 1.45]	.037^*^	.04 (.34)	[–.62; .70]	.911	.66 (.33)	[.02; 1.30]	.043^*^
Maternal education	.18 (.25)	[–.31; .66]	.481	.15 (.21)	[–.27; .57]	.480	.14 (.25)	[–.34; .63]	.563
Time between assessments	–.03 (.02)	[–.07; .01]	.187	.02 (.02)	[–.03; .06]	.504	–.02 (.03)	[–.07; .03]	.501
*R* ^2^	.12 (.10)		.240	.33^***^ (.09)		.000	.32^†^ (.20)		.106
**Step 3**									
Pre‐adoptive RF				.56 (.10)	[.37; .75]	.000	.08 (.19)	[–.29; .44]	.675
Maternal RF^a^							.38 (.27)	[–.15; .92]	.162
Child gender^a^	.75 (.36)	[.05; 1.45]	.037^*^	.04 (.34)	[–.62; .70]	.905	.62 (.31)	[.01; 1.24]	.048^*^
Maternal education	.18 (.25)	[–.31; .66]	.481	.15 (.21)	[–.27; .57]	.478	.13 (.24)	[–.35; .60]	.605
Time between assessments	–.03 (.02)	[–.07; .01]	.187	.02 (.02)	[–.03; .06]	.511	–.02 (.03)	[–.07; .04]	.609
*R* ^2^	.12 (.10)		.240	.34^***^ (.09)		.000	.31 (.19)		.111

*Note*: Standardized coefficients are presented.

Abbreviation: RF, reflective functioning.

^a^Male versus female.

^†^ *p* < .10.

* *p* < .05.

*** *p* < .001.

#### 3.2.2 The predictive role of paternal reflective functioning

**FIGURE 3:**
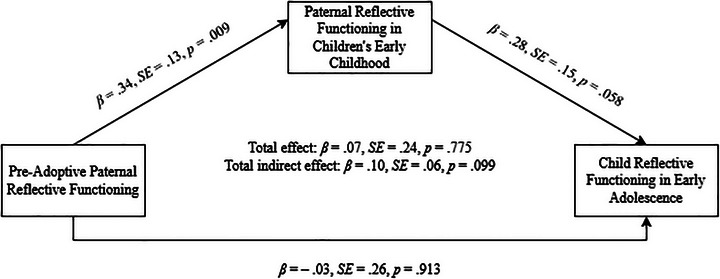
Final paternal mediation model.

The models for paternal reflective functioning showed a perfect model fit; full results are presented in Table 5. In Step 1, pre‐adoptive paternal reflective functioning had no direct effect on child reflective functioning (*β* = .04, *SE* = .24, *p* = .858). In Step 2, pre‐adoptive paternal reflective functioning significantly predicted paternal reflective functioning in the adopted child's early childhood (*β* = .34, *SE* = .13, *p* = .009), which predicted child reflective functioning in adolescence (*β* = .28, *SE* = .14, *p* = .046). The total indirect effect approached significance (*β* = .09, *SE* = .05, *p* = .073). In Step 3, the direct effect from pre‐adoptive paternal reflective functioning to child reflective functioning was nonsignificant (*β* = –.03, *SE* = .26, *p* = .913), although the link between paternal reflective functioning in early childhood and child reflective functioning in adolescence trended toward significance (*β* = .28, *SE* = .15, *p* = .058) (Figure 3). The total effect was nonsignificant (*β* = .07, *SE* = .24, *p* = .775), but the total indirect effect approached significance (*β* = .10, *SE* = .06, *p* = .099). Finally, a model comparison showed no significant improvement with the direct path (ΔSBS‐*χ* ^2^(1) = .01, *p* = .913).

**TABLE 5:** Mediation model of paternal reflective functioning.

	Predictor Pre‐adoptive RF			Mediator Paternal RF			Outcome Child RF		
	*β* (*SE*)	95% CI	*p*‐value	*β* (*SE*)	95% CI	*p*‐value	*β* (*SE*)	95% CI	*p*‐value
**Step 1**									
Pre‐adoptive RF							.04 (.24)	[–.43; .51]	.858
Child gender^a^	.34 (.37)	[–.38; 1.07]	.352				.68^†^ (.40)	[–.10; 1.47]	.089
Paternal education	.52 (.17)	[.19; .86]	.002				.26 (.21)	[–.15; .66]	.217
Time between assessments	.00 (.03)	[–.05; .06]	.899				–.03 (.03)	[–.08; .02]	.290
*R* ^2^	.17^†^ (.10)		.077				.15 (.12)		.228
**Step 2**									
Pre‐adoptive RF				.34^**^ (.13)	[.09; .59]	.009			
Paternal RF							.28^*^ (.14)	[.01; .55]	.046
Child gender^a^	.34 (.37)	[–.38; 1.07]	.352	–.41 (.28)	[–.96; .13]	.138	.86^*^ (.38)	[.12; 1.60]	.023
Paternal education	.52^**^ (.17)	[.19; .86]	.002	.13 (.21)	[–.28; .54]	.538	.17 (.19)	[–.21; .55]	.374
Time between assessments	.00 (.03)	[–05; .06]	.899	.01 (.04)	[–.06; .08]	.801	–.03 (.02)	[–.07; .02]	.199
*R* ^2^	.17^†^ (.10)		.077	.19^†^ (.10)		.053	.23^†^ (.12)		.059
**Step 3**									
Pre‐adoptive RF				.34^**^ (.13)	[.09; .59]	.009	–.03 (.26)	[–.53; .48]	.913
Paternal RF							.28^†^ (.15)	[–.01; .58]	.058
Child gender^a^	.34 (.37)	[–.38; 1.07]	.352	–.41 (.28)	[–.96; .13]	.138	.87^*^ (.39)	[.11; 1.63]	.024
Paternal education	.52^**^ (.17)	[.19; .86]	.002	.13 (.21)	[–.28; .54]	.538	.18 (.20)	[–.20; .57]	.355
Time between assessments	.00 (.03)	[–.05; .06]	.899	.01 (.04)	[–.06; .08]	.801	–.03 (.02)	[–.07; .02]	.195
*R* ^2^	.17^†^ (.10)		.077	.19^†^ (.10)		.053	.23^†^ (.12)		.058

*Note*: Standardized coefficients are presented.

Abbreviation: RF, reflective functioning.

^a^Male versus female.

^†^ *p* < .10.

* *p* < .05.

** *p* < .01.

#### 3.2.3 Socioemotional difficulties

##### 3.2.3.1 Longitudinal associations with parents’ reflective functioning

Analyses showed that neither maternal (*β* = –.23, *SE* = .20, *p* = .239) nor paternal (*β* = .18, *SE* = .23, *p* = .437) pre‐adoptive reflective functioning predicted internalizing adolescent difficulties. For externalizing difficulties, there was a trend toward significance for maternal pre‐adoptive reflective functioning (*β* = –.39, *SE* = .20, *p* = .054), but not for paternal pre‐adoptive reflective functioning (*β* = –.22, *SE* = .22, *p* = .326). When examining parental reflective functioning in the adoptive child's early childhood, maternal reflective functioning was not associated with internalizing difficulties (*β* = .22, *SE* = .25, *p* = .376), whereas paternal reflective functioning was positively associated with internalizing difficulties (*β* = .38, *SE* = .17, *p* = .024). However, after applying the Benjamini–Hochberg correction for multiple comparisons, the association between paternal reflective functioning and internalizing difficulties was no longer statistically significant (corrected *p*‐value threshold = .005). For externalizing difficulties, neither the association with maternal reflective functioning (*β* = .17, *SE* = .23, *p* = .449) nor the association with paternal reflective functioning (*β* = .05, *SE* = .16, *p* = .771) was significant.

##### 3.2.3.2 Cross‐sectional associations with adolescents’ reflective functioning

Child reflective functioning was not significantly associated with internalizing difficulties as reported by adolescents (*β* = .35, *SE* = .26, *p* = .181), or adoptive mothers (*β* = .21, *SE* = .15, *p* = .151) or adoptive fathers (*β* = .11, *SE* = .25, *p* = .642). Similarly, child reflective functioning showed no significant associations with externalizing difficulties, either as self‐reported by adolescents (*β* = –.01, *SE* = .25, *p* = .973) or as reported by adoptive mothers (*β* = .09, *SE* = .18, *p* = .619) or adoptive fathers (*β* = –.19, *SE* = .20, *p* = .338).

### 3.3 Sensitivity analyses

#### 3.3.1 Moderating role of child age at placement

**FIGURE 4:**
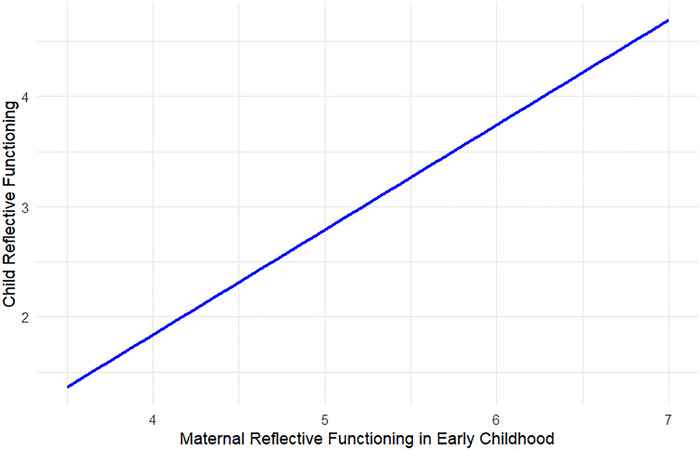
Moderation of child age at placement in the association between maternal reflective functioning and child reflective functioning. Child age at placement is measured in months (*M* = 13.43, *SD* = 6.56, range = 4–30).

Results indicated that child age at placement significantly moderated the association between maternal reflective functioning in the adopted child's early childhood and child reflective functioning in adolescence (*β* = –.07, *SE* = .03, *p* = .040), suggesting that the association between maternal and child reflective functioning was attenuated by an increase in child age at placement (Figure 4). No moderation effect was found between pre‐adoptive maternal reflective functioning and maternal reflective functioning in early childhood (*β* = .02, *SE* = .03, *p* = .436), but a trend appeared for pre‐adoptive maternal reflective functioning and child reflective functioning (*β* = .13, *SE* = .07, *p* = .062). For adoptive fathers, there was a trend for child age at placement to moderate the paternal–child reflective functioning association (*β* = .04, *SE* = .02, *p* = .053). No significant moderation effects or trends were found for other associations.

**FIGURE 5:**
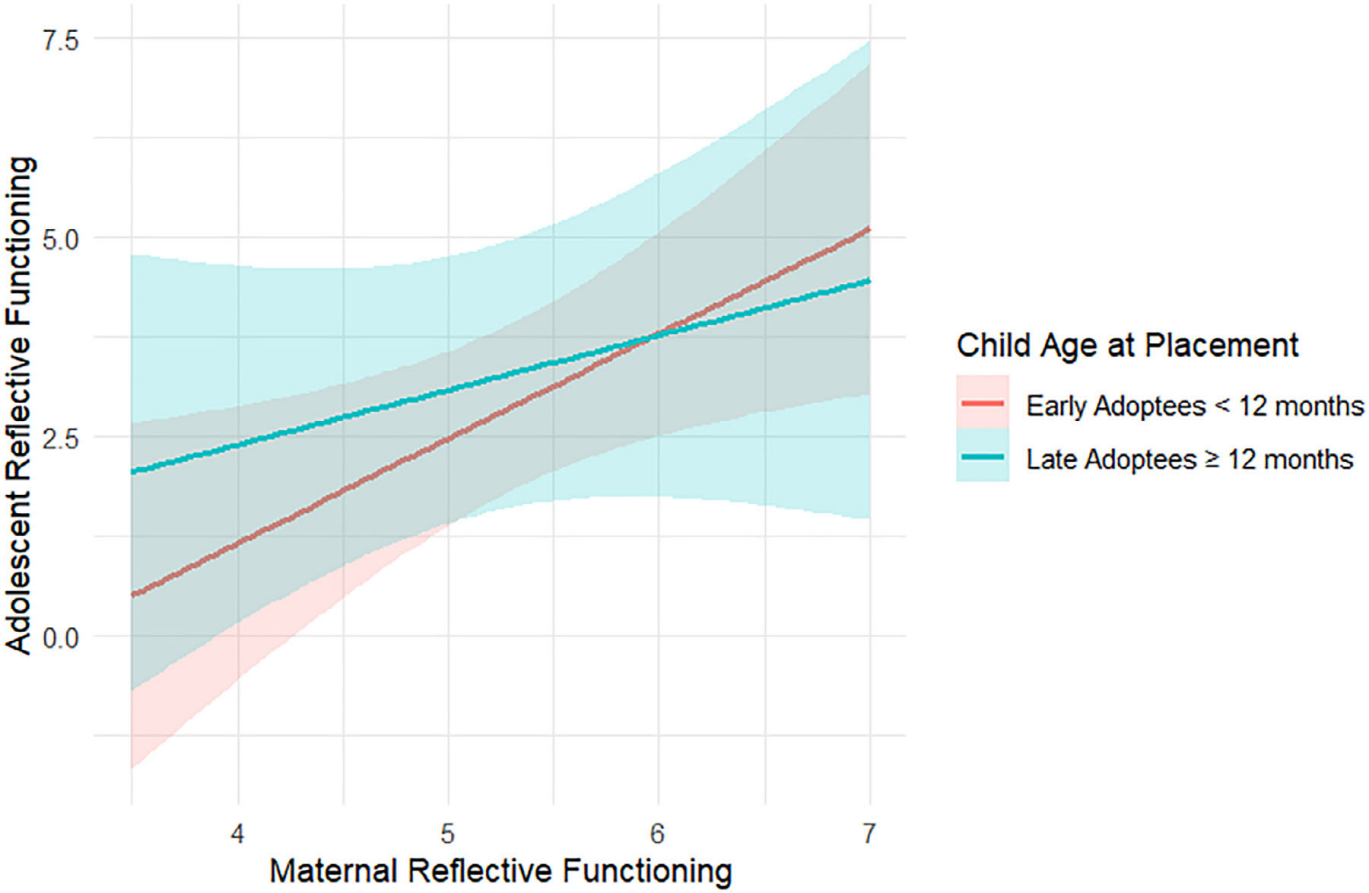
Moderation of child age at placement using a cut‐off of 12 months in the association between maternal reflective functioning and child reflective functioning.

To investigate the moderating role of child age at placement using the cut‐off of 12 months, multigroup analyses were conducted. Pre‐adoptive reflective functioning was associated with maternal reflective functioning in early childhood for both early (<12 months at placement) (*β* = .56, *SE* = .11, *p* < .001) and late (≥12 months at placement) adoptees (*β* = .48, *SE* = .12, *p* < .001), but did not predict child reflective functioning in either group. Maternal reflective functioning in early childhood significantly predicted child reflective functioning in adolescents who had been early adoptees (*β* = .87, *SE* = .26, *p* = .001), but did not significantly predict child reflective functioning in those who had been late adoptees (*β* = .14, *SE* = .33, *p* = .672) (Figure 5). For adoptive fathers, pre‐adoptive reflective functioning predicted paternal reflective functioning in early childhood among late adoptees (*β* = .34, *SE* = .15, *p* = .025) and trended toward significance for early adoptees (*β* = .28, *SE* = .16, *p* = .075).

#### 3.3.2 Monte Carlo post hoc power analysis

In the maternal model, the path from pre‐adoptive reflective functioning to maternal reflective functioning in early childhood had excellent power (1.00). However, the path from pre‐adoptive reflective functioning to child reflective functioning in early adolescence had low power (.086), suggesting a higher risk of Type II error. The path from maternal reflective functioning in early childhood to child reflective functioning in early adolescence showed a slightly underpowered path (.740) but indicated a reasonable likelihood of detecting an effect. In the paternal model, the paths from pre‐adoptive reflective functioning to paternal reflective functioning in early childhood (.987) and from paternal reflective functioning in early childhood to child reflective functioning in early adolescence (.992) had strong power. However, the direct path from pre‐adoptive reflective functioning to child reflective functioning was underpowered (.080).

## 4 DISCUSSION

The present study investigated whether pre‐adoptive reflective functioning in adoptive mothers and fathers, together with parental reflective functioning in their adopted child's early childhood, predicted child reflective functioning and socioemotional difficulties in transnationally adopted children in early adolescence when children were on average 12 years old. Both maternal and paternal pre‐adoptive reflective functioning predicted parental reflective functioning in the adopted child's early childhood. Furthermore, both maternal and paternal reflective functioning in early childhood, but not pre‐adoptive reflective functioning, predicted child reflective functioning in early adolescence. The findings indicate that parental reflective functioning in relation to one's actual child shapes adolescents' reflective capacities regarding themselves and their relationships with their attachment figures. Against expectations, there were no longitudinal associations between parents’ reflective functioning and socioemotional difficulties in adolescence. The following sections will discuss the findings of this study and their implications.

### 4.1 Stability of reflective functioning in the transition to adoptive parenthood

First, findings indicated that pre‐adoptive reflective functioning was positively correlated with parental reflective functioning in the adopted child's early childhood among adoptive mothers. This is consistent with previous findings in non‐adoptive parents where correlations were found in reflective functioning about an imagined child assessed during pregnancy and parental reflective functioning about their actual child in early childhood, with small to medium effect sizes (Crumbley, 2009; Pajulo et al., 2012; Wong, 2016). At the same time, reflective functioning measured after placement about the actual adopted child was lower than reflective functioning about their imagined child before placement. This was particularly pronounced among adoptive fathers. A closer investigation of the scores indicated that a substantial group of parents retained similar scores in the transition to adoptive parenthood, whereas a subgroup showed significantly lower reflective functioning scores about the actual adopted child. There are a few possible explanations for these findings. As Slade and Sleed (2024) formulated it, parental reflective functioning is assessed in the context of an interview probing the parent to reflect upon a “live” and “hot” relationship with the child. Possibly, it is inherently more challenging for parents, in the context of both biological and adoptive parenthood, to reflect upon the mental states of an actual child after birth or adoption.

Second, the finding that fathers scored lower than mothers in reflective functioning when considering the mental states of their actual child raises the question of whether stability in reflective functioning differs between adoptive mothers and fathers, or between mothers and fathers more generally. One could consider this finding in light of a growing body of evidence suggesting that some stressors may be more pronounced for fathers in their transition to parenthood (Molloy et al., 2022), and that stress has been found to put reflective functioning under pressure (Luyten et al., 2020). For example, research has demonstrated that fathers experience elevated stress levels related to social isolation (Philpott et al., 2017). Likewise, declines in relationship satisfaction, common to both mothers and fathers during the transition to parenthood, tend to be more pronounced among fathers and are linked to increased stress (Don & Mickelson, 2014; ter Kuile et al., 2021). In the specific context of transnational adoption, studies have indicated that stress spills over into the marital relationship among adoptive fathers, but not among adoptive mothers, and that this spillover is significantly associated with lower reflective functioning (Malcorps et al., 2022). Nevertheless, further research is needed to examine these processes more thoroughly, especially because differences in reflective functioning may be explained by other factors as well. Although gender roles are changing rapidly, it could also be that adoptive fathers may still spend less time with their children compared with mothers, which could provide them with fewer opportunities to develop a more nuanced understanding of the child (Lamb, 2010). At the same time, previous studies have found that women generally score higher on mentalizing measures, likely due to biological, social, and cultural factors (Proverbio, 2023; Smeets et al., 2009), and that, in Flanders at least, selection procedures among adoptive parents tend to result in a particularly high‑functioning group of mothers, who remain relatively stable even under stress (Fiore et al., 2025).

### 4.2 Intergenerational transmission of reflective functioning

As hypothesized, results indicated that the parental reflective functioning of adoptive mothers and fathers in early childhood predicted child reflective functioning in early adolescence, suggesting moderate effect sizes and adding longitudinal evidence to the notion of the intergenerational transmission of reflective functioning (Rosso & Airaldi, 2016). Pre‐adoptive reflective functioning was not significantly associated with child reflective functioning, suggesting that it was not reflective functioning assessed prior to adoption, but parental reflective functioning in relation to a real child shortly after adoption, that facilitates the development of child reflective functioning. This distinction may highlight a key complexity of adoption: reflective functioning prior to placement may reflect adoptive parents’ hopes, ideals and fantasies. However, once the actual child has joined the family, adoptive parents are confronted with the realities of their child's individual temperament, history, and potential socioemotional difficulties. It is within this demanding and sometimes destabilizing context, referred to as a period of “post‐adoption blues” (Foli & Thompson, 2004; Foli et al., 2017), that the role of parental reflective functioning may become even more meaningful. Rather than reflective functioning prior to adoption, it is the adoptive parents’ ability to reflect on and integrate challenging experiences often inherent to adoption that predicts the child's reflective functioning.

This study provides evidence for a longitudinal association between parents’ reflective capacities in early childhood and those of their children more than a decade later in adolescence, thereby adding support to the notion of intergenerational transmission of reflective functioning. At the same time, children's reflective capacities continue to develop across contexts and relationships, and parents’ early reflective functioning is best understood as an early indicator of their ongoing ability to flexibly attune to both their own and their child's mental states throughout development. For example, parents of infants may rely primarily on their reflective capacities to identify the sources of their infant's discomfort and to regulate the infant's affective states (Campbell et al., 2024), whereas parents of preschoolers may be more likely to foster pretend play and make‐believe based on their understanding of their child's mental states (Ensink & Mayes, 2010). In parents of toddlers, reflective capacities could, for instance, support developmental themes such as sharing, taking turns, and “togetherness”, where parents can explicitly explain the perspective of others to the child (Degotardi et al., 2014). From school age onward, parents can scaffold the cognitive and socioemotional development of their child's reflective capacities by expressing their understanding of and genuine interest in their child's mental states in everyday life.

### 4.3 Child age at placement: Implications for transmission of maternal and child reflective functioning

Child age at placement (as a proxy for early adversity) moderated the association between maternal reflective functioning in early childhood and child reflective functioning in adolescence, but did not moderate the association between pre‐adoptive reflective functioning and parental reflective functioning in early childhood. In both contexts, some hypotheses on the role of child age at placement and child‐related factors more generally are justifiable in light of the growing evidence for the role of child age at placement as a proxy of early adversity (Fiore et al., 2025; Julian, 2013; Malcorps et al., 2022).

Regarding the presence of a moderation effect in the association between maternal reflective functioning in early childhood and child reflective functioning in adolescence, it is plausible that children placed at older ages may have experienced prolonged periods of adversity, which could have long‐term consequences for the development of their reflective capacities. Whereas nurturing interactions after adoption have been shown to buffer the effects of early adversity on children's socioemotional development (Paine et al., 2021), early adversity has been found to alter the stress system in important ways, potentially increasing the child's vulnerability to difficulties later in life (McCrory et al., 2017). Moreover, adversity may exert not only a direct effect on the socioemotional development of children, but also an indirect one. Adoptive parents of children adopted at a later age, after the child may have experienced periods of prolonged adversity, may encounter additional challenges in regulating their child's affective states and understanding the mental states underlying behaviors related to past traumatic experiences (e.g., an adopted infant may panic with overwhelming intensity when briefly left alone, a reaction that goes beyond normative separation anxiety and may reflect earlier experiences of abandonment). This could complicate the child's experience of feeling recognized and hinder parents in offering second‐order representations of their child's mental states. However, although early adversity can present profound challenges, it is essential to maintain a balanced perspective and recognize both the potential negative effects of adversity and the capacity for positive change within a nurturing environment that recognizes the plasticity of children's development (Belsky & Pluess, 2013). Therefore, more research is needed to investigate the interplay between risk and resilience processes.

The absence of a moderating effect of child age at placement in the association between parents’ reflective capacities prior to adoption and in early childhood may reflect several factors. At 6 months post‐placement, parents are typically still in a phase characterized by hope, engagement, and positive bonding with their child, which may buffer the immediate impact of early adversity on parental reflective functioning. In addition, the child's challenges may not yet have fully emerged, so potential effects of early adversity on parental reflective capacities might become apparent only later in development. Methodological aspects of the RFS may also limit the detection of early changes. In particular, hypermentalizing—a maladaptive pattern in which parents make excessive or inaccurate inferences about a child's mental states beyond what can reasonably be inferred from behavior—can be difficult to distinguish from genuinely adaptive reflective functioning (Sharp et al., 2013), as discussed in a recent narrative review on the RFS (Slade & Sleed, 2024). As a result, early post‐placement assessments may appear stable, even though some parents may already be struggling to understand their child's mental states in ways that reflect emerging difficulties. This process may be especially relevant for parents of older adopted children, who are more likely to present complex behavioral and socioemotional difficulties. In this regard, a recent study found that prementalizing among adoptive fathers increased during the first 4 years post‐adoption, but only for children placed after 18 months, an increase that was positively associated with children's socioemotional difficulties (Malcorps et al., 2022).

### 4.4 Socioemotional adolescent difficulties

Overall, most adoptive parents and adolescents reported socioemotional difficulties within the normative range in early adolescence, indicating that many adolescents were well‐functioning. Nevertheless, a notable and pronounced subgroup exhibited elevated difficulties, particularly in the externalizing domain, suggesting a heightened risk for externalizing difficulties. No significant differences were observed between adoptive mothers’ and fathers’ reports. Beyond child age at placement, there were no consistent sex differences, with the exception that boys self‐reported slightly higher internalizing symptoms than girls. As internalizing difficulties are generally more prevalent among girls (Costello et al., 2003), these findings should be interpreted cautiously, as the study was not sufficiently powered to examine sex‐specific pathways, underscoring the need for further research on the role of child sex in the socioemotional development of transnational adoptees.

### 4.5 Limitations and strengths

This study extends the existing literature in two important ways. First, it examines the intergenerational transmission of reflective functioning using a longitudinal design spanning more than 10 years, providing new evidence on how parental reflective capacities associate with the development of adopted children's reflective functioning in adolescence. Second, by assessing adoptive mothers’ and fathers’ reflective capacities both before and after child placement, the study addresses the stability of reflective functioning during the transition to adoptive parenthood, a topic that, to date, has not been systematically investigated in adoptive families.

The study also has some limitations. The primary limitation concerns the small sample size, which increases the risk of Type II errors and constrains the ability to detect small to medium effects, particularly in analyses involving moderators. Although the robustness of the findings is supported by the availability of a clear theoretical framework, the growing cross‐sectional evidence in this area, and post hoc power analyses using a Monte Carlo approach, further research with larger sample sizes is needed. Another limitation is that the present study relied on child age at placement as a proxy for early adversity, rather than using more direct measures. A final limitation is that the study did not assess parental reflective functioning in early adolescence, which could shed light on potential differences in the strength of prospective and cross‐sectional associations.

## 5 CONCLUSION

There is increasing evidence that parents’ reflective functioning significantly influences the development of reflective functioning in their children, underscoring the concept of intergenerational transmission of reflective functioning. To address the gap in longitudinal evidence, this study prospectively examined associations between reflective functioning during the transition to adoptive parenthood, parental reflective functioning in adopted children's early childhood, and adopted children's reflective functioning in early adolescence. Findings indicated that pre‐adoptive reflective functioning predicted parental reflective functioning in early childhood. Furthermore, a key contribution of this study is that parental reflective functioning in early childhood predicted the reflective functioning of adopted children in early adolescence. However, parental reflective functioning was not associated with socioemotional adolescent difficulties in this adoptive sample. Taken together, these findings provide longitudinal support for the intergenerational transmission of reflective functioning, while also highlighting the need for a broader exploration of other factors influencing socioemotional difficulties in adolescence in the context of adoption.

## CONFLICT OF INTEREST STATEMENT

The authors have no financial or proprietary interests in any material discussed in this article.

## CONSENT TO PARTICIPATE

Written informed consent was obtained from all individual participants included in the study.

## Data Availability

Given that this study focuses on families and children who are sometimes at increased risk for the development of socio‐emotional difficulties, and it is a relatively small sample, increasing the risk that other researchers might identify the participants, in principle, we will not share any data (including coded data). We will consider requests for data sharing/access only if researchers can fully guarantee participants’ confidentiality and anonymity.
